# Urgent Notification Intervention of Home Blood Pressure in Cohort Studies of the Tohoku Medical Megabank Project

**DOI:** 10.31662/jmaj.2023-0215

**Published:** 2024-07-08

**Authors:** Eiichi N Kodama, Makiko Taira, Hideyasu Kiyomoto, Tomohiro Nakamura, Satoshi Nagaie, Shinichi Kuriyama, Atsushi Hozawa, Junichi Sugawara, Fuji Nagami, Akira Uruno, Jun Nakaya, Hirohito Metoki, Masaki Sakaida, Masahiro Kikuya, Yoichi Suzuki, Kiyoshi Ito, Yohei Hamanaka, Kichiya Suzuki, Shigeo Kure, Nobuo Yaegashi, Nobuo Fuse, Ritsuko Shimizu, Masayuki Yamamoto

**Affiliations:** 1 Tohoku Medical Megabank Organization, Tohoku University, Sendai, Japan; 2 Graduate School of Medicine, Tohoku University, Sendai, Japan; 3 Tohoku University Hospital, Tohoku University, Sendai, Japan; 4 International Research Institute of Disaster Science, Tohoku University, Sendai, Japan; 5 Faculty of Data Science, Kyoto Women’s University, Kyoto, Japan; 6 Suzuki Memorial Hospital, Iwanuma, Japan; 7 Graduate School of Medicine, Hokkaido University, Sapporo, Japan; 8 Tohoku Medical and Pharmaceutical University, Sendai, Japan; 9 Teikyo University School of Medicine, Tokyo, Japan; 10 Ageo Central General Hospital, Ageo, Japan; 11 Cancer Detection Center, Miyagi Cancer Society, Sendai, Japan; 12 Miyagi Children’s Hospital, Sendai, Japan; 13 Advanced Research Center for Innovations in Next-Generation Medicine, Tohoku University, Sendai, Japan

**Keywords:** urgent notification, Tohoku Medical Megabank Project, blood test, home blood pressure

## Abstract

**Introduction:**

The Tohoku Medical Megabank (TMM) was established for creative reconstruction from the Great East Japan Earthquake and tsunami in 2011. Two prospective genome cohort studies in Miyagi prefecture have successfully recruited approximately 127,000 participants. The health status of these individuals was evaluated at the initial recruitment, and follow-up health checkups have been conducted every 5 years. During these health checkups, unexpected critical values were encountered, which prompted us to develop an urgent notification system.

**Methods:**

We analyzed the frequency of critical values observed in home blood pressure (HBP) test in an urgent notification office (UNO). We returned the critical values by urgent notification before the notifications of regular results. In addition, the impact of the TMM urgent notification on the participants was evaluated.

**Results:**

We issued urgent notifications of the critical values of extremely high HBP. Of the 21,061 participants who underwent HBP measurements, 256 (1.2%) met the criteria for urgent notification. It was found that abnormalities in blood sugar levels, renal function, and lipid values were frequently concurrent with the abnormal HBP readings. Annual questionnaires administered after the urgent notification, approximately 60% of those went to hospitals or clinics.

**Conclusions:**

The urgent notification system for hypertensive emergency with HBP in the TMM was well accepted by the participants and encouraged them to seek medical care. The system has been useful in addressing the prolonged healthcare problems and in promoting health care in large-scale disaster damaged areas.

## Introduction

The Tohoku Medical Megabank (TMM) was established for reconstruction from the Great East Japan Earthquake (GEJE) and tsunami, which caused great damage to the northern pacific coast of Japan ^(1)^. The TMM comprises two integral organizations at Tohoku University and Iwate Medical University, referred to as ToMMo ^(2), (3)^ and IMM ^(4)^, respectively. ToMMo focuses on the survey of postdisaster health situations in Miyagi prefecture after the GEJE and contributes to future personalized healthcare and medicine by establishing prospective genome cohorts and a biobank ^(3), (5), (6), (7), (8), (9), (10), (11), (12)^.

Large-scale population cohort studies usually minimize interventions and excessive exposures to participants. Conversely, returning the health checkup results to participants is beneficial, making them knowledgeable of their health status and contributing to an overall improvement in their well-being. Therefore, considering the purpose of TMM that works on the survey of postdisaster health situations, the TMM made a conscious decision to return health checkup results to the participants ^(8)^. For this purpose, ToMMo covering the participants in Miyagi prefecture has established a system returning health checkup results after the admission ^(6), (8)^.

Our health checkup occasionally identifies participants with critically abnormal values or critical values. Therefore, in addition to the regular health check notification system, ToMMo developed an urgent notification system that informs participants about their abnormal results within 1 week after the identification of the critical values. We believe that this system will contribute to the maintenance of the participants’ health. To the best of our knowledge, the urgent notification system is uncommon in prospective cohort studies. Therefore, the design and operation of the urgent notification system coupled with the return of ordinary results have been a fascinating challenge for ToMMo.

This study describes three critical features in the design and operation of the urgent notification system of ToMMo for hypertension. First, we selected critical values of home blood pressure (HBP) test among the various health checkups for the urgent notification, as it is medically important to notify participants when their high HBP is detected. Second, we rigorously established selection criteria for participants who should receive urgent notification. Lastly, we evaluated the impacts of urgent notification on the participants’ behaviors. By elucidating these three aspects, we aimed to shed light on the significance of urgent notification for HBP.

## Materials and Methods

### Study design and population

Critical values for urgent notifications were screened while conducting health checkups of the TMM Community-Based Cohort Study (TMM CommCohort Study) ^(8)^ and Birth and Three-Generation Cohort Study (TMM BirThree Cohort Study) ^(7), (13), (14)^. Details of the health checkups were published elsewhere ^(6), (7)^. This study included male and female patients aged at least 20 years who were living in Miyagi prefecture in Japan. The survey and recruitment were conducted between May 2013 and March 2016. Informed consent or ascent was obtained from the participants. This study was approved by the Institutional Review Board of the ToMMo (approval number: 2022-4-047; approval date: June 30, 2022).

We excluded the participants who withdrew consent by December 21, 2021 from total participants (n = 13,855) who underwent HBP measurements. Finally, 12,523 participants (3,728 men and 8,795 women) were analyzed. Those who withdrew consent by December 21, 2021, were excluded. Finally, 12,523 participants (3,728 men and 8,795 women) were analyzed.

### Home blood pressure

HBP was assessed using a cuff oscillometric device (HEM-7080IC; Omron Healthcare Co., Ltd.), which has been approved as a reliable equipment in Japan. The measurements were recorded for 14 days and subsequently analyzed. The hypertension criteria for conventional blood pressure of the 2009 Japanese Society of Hypertension (JSH2009) guidelines ^(15)^ were generally applied to our HBP survey.

### Other measurements

Basic information including blood and urine laboratory test data was used as reference as previously described ^(3), (6), (7)^. Nonfasting blood samples were obtained. The normal value ranges are listed in Table 1. The results of the health examination were regularly returned to the participants. A self-reported questionnaire was used to assess the patients’ demographic characteristics, smoking and drinking status, educational level, physical activity, and history of respiratory disease ^(6)^. Age was determined at the time of visit to the community support center.

**Table 1.** Limits of Blood Test Values Used in ToMMo.

Test name	Normal ranges
CRP (mg/dL)	≤0.30
Hemoglobin (g/dL)	Male: 13.5-17.5 Female: 11.5-15.0
Platelet count (10^4^/μL)	14.0-34.0
Leukocyte count (/μL)	3,300-9,000
Abnormal cells	Presence of immature blast cells, multiple atypical cells, or more than 30 erythroblasts in 200 nucleated cells
Blood sugar (mg/dL)	70-109
AST (GOT) (IU/L)	10-40
ALT (GPT) (IU/L)	5-45
Creatinine (mg/dL)	Male: 0.61-1.04 Female: 0.47-0.79
Blood urea nitrogen (mg/dL)	8.0-20.0
Uric acid (mg/dL)	Male: 3.8-7.0 Female: 2.5-7.0
Serum Na (mEq/L)	137-147

The recommended values for normal ranges from two clinical testing companies, Bio Medical Laboratories, Inc., Sendai, Japan, and LSI Medience, Sendai, Japan, were used as our reference ranges for normal values.

### Statistical analysis

To analyze the relation of two or more abnormal values, Pearson’s chi-squared test or Fisher’s exact test was conducted on the number of participants in 2 × 2 tables. The calculated *P*-values are also included in Table 2. Bonferroni-adjusted significance level (α) was set to 0.05/10 (0.005). *P* < 0.005 was considered to indicate statistical significance. Any changes resulting from urgent notification letters in receiving medical care for hypertension were analyzed using McNemar’s chi-squared test. All analyses were conducted using the R software version 4.3.2 (R Core Team, Vienna, Austria).

**Table 2.** Summary of Accompanying Abnormal Finding in Health Examination in Participants Receiving Home Blood Pressure Urgent Notification.

		Renal dysfunction		Lipid abnormality		Hematological abnormality		Liver dysfunction	
		+	-	+	-	+	-	+	-
Abnormal Blood sugar	+ (n = 118)	51	67	85	33	17	101	18	100
	- (n = 136)	50	86	5	131	25	111	10	126
	p-value	0.3576^1^		<0.0001^2^		0.4957^1^		0.07115^1^	
Renal dysfunction	+ (n = 101)			40	61	19	82	12	89
	- (n = 153)			50	103	23	130	16	137
	p-value			0.3197^1^		0.5347^1^		0.8809^1^	
Lipid abnormality	+ (n = 90)					11	79	13	77
	- (n = 164)					31	133	15	149
	p-value					0.2324^1^		0.2801^1^	
Hematological abnormality	+ (n = 42)							3	39
	- (n = 212)							25	187
	p-value							0.5889^2^	
Liver dysfunction	+ (n = 28)
	- (n = 226)
	p-value

The number of participants is shown in the table. The symbols “+” and “−” in the column and raw labels indicate “presence” and “absence” of the abnormalities, respectively. Statistical analyses of participant count in 2 × 2 tables were conducted using ^1^Pearson’s chi-squared test or ^2^Fisher’s exact test, and the corresponding *P*-values were calculated.

## Results

### Information management for TMM cohort studies

**Figure 1.**
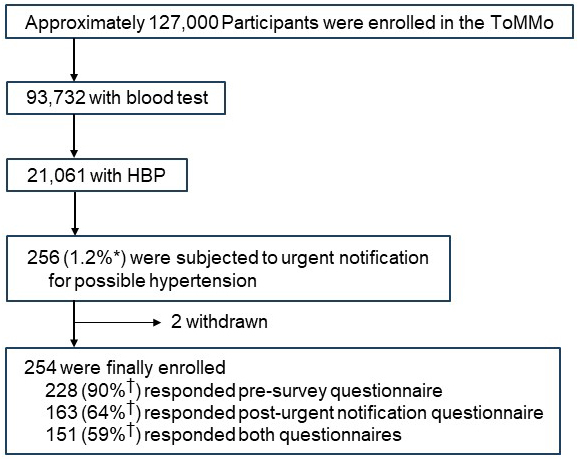
Eligibility and enrollment of participants for home blood pressure. The percentage shown was calculated based on the number of participants for home blood pressure (HBP) measurement (n = 21,061)^*^ and that of participants who received urgent notification letters (excluding 2 withdrawn participants, n = 254)^†^.

Participant recruitment was conducted from May 2013 to May 2017 in Miyagi prefecture ^(6), (7), (8)^. Of the participants, approximately 127,000 (as of June 1, 2023) were recruited in Miyagi prefecture by ToMMo (Figure 1). These individuals were recruited either on the sites for specific health checkups for the annual community health examination conducted by the municipal organization or through direct visits for health examination to the community support centers located at seven sites in Miyagi prefecture ^(3)^. Blood samples were obtained from 93,732 (as of June 1, 2023) individuals who participated in the TMM CommCohort Study and TMM BirThree Cohort Study.

### HBP monitoring in TMM

**Figure 2.**
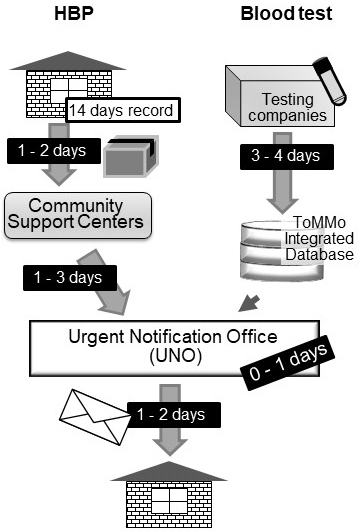
Diagram of operation time required in each step. Reports showing high values of blood test and home blood pressure (HBP) were transferred to the urgent notification office (UNO) from the ToMMo integrated database and community support centers, respectively. After assessments, an urgent notification letter was sent via mail to the participant, if necessary. The urgent notification letters arrived at the participants’ address within1 week after return of the sphygmomanometer device to the community support centers.

In addition to conducting blood tests, we monitored the HBP of the participants aged >20 years who were undergoing health examination at our seven community support centers (Figure 2) ^(6)^. Participants aged under 19 years were also included in the HBP monitoring, if they attended in the BirThree Cohort Study as parents. Eventually, 21,061 participants were subjected to HBP monitoring using automated sphygmomanometers supplied by ToMMo (Figure 1).

The participants were instructed to measure their HBP every day, at least once in the morning and once in the evening for 14 days. Any additional measurements during midday/afternoon were appreciated. The morning, midday/afternoon, and evening time periods were defined as 4:00 AM-11:00 AM, 11:00 AM-4:00 PM, and 4:00 PM-4:00 AM of the next day, respectively. The devices were returned to our community support centers after the monitoring (Figure 2). Subsequently, the measurements were extracted from the device and analyzed within 1-3 days after the return of the device. In the case where the participants measured their HBP two or more times within the same time period on the same day, the first measurement value was used. The average values of HBP in each time period were determined for 14 days. When the average HBP value in any time periods was greater than the urgent notification criterion, the information was immediately notified to the UNO.

### Algorithm for urgent notification of blood pressure results

**Figure 3.**
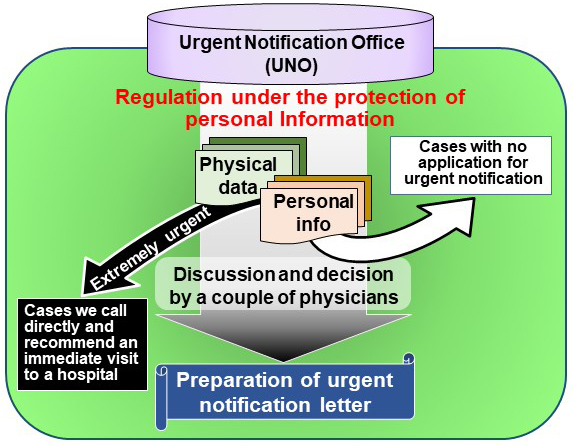
Workflow of the urgent notification system. The decision on whether to send an urgent notification letter to a participant was made by the UNO, where several physicians and a specialist comprehensively discussed all the data available in our baseline assessment, including other inspection results, medical history, and medication records. If a decision was made to return the data, an urgent notification letter was prepared within 1 business day, and the letter was confirmed by multiple UNO members.

We designed a system in which we can return abnormal values in the blood test and HBP to the patients. In this system, if there are abnormal values in the blood test or HBP, a notification will be immediately sent to urgent notification office (UNO), which comprises several physicians and a specialist. The activities of the UNO are summarized in Figure 3. After receiving the data, more than two UNO members available at the time discuss the data and decide whether the data should be returned to the participants. If it is decided to return the data immediately, an urgent notification letter is prepared. Before being sent out, the letter is confirmed by multiple UNO members.

During the baseline health assessments from May 2013 to May 2017, 297 reports of suspicious cases of hypertensive emergency were reviewed. Upon making the decision in UNO (Figure 3), all available data in our baseline assessment were comprehensively considered, which included other inspection results, medical history, and medication records. We sent an urgent notification letter within 0-1 business day after the information was received by UNO (Figure 2). The participants were directly contacted to inquire about their medical conditions as needed.

**Figure 4.**
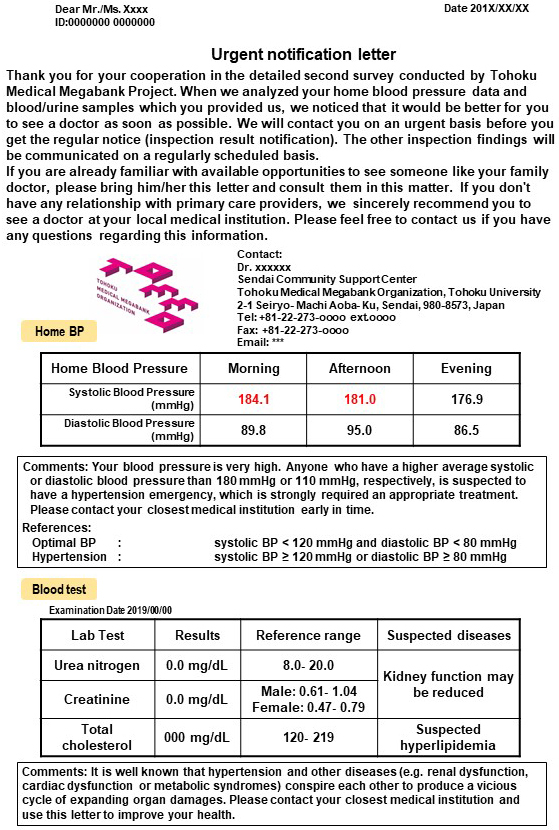
Example of the urgent notification letter sent to participants with abnormal home blood pressure value. In addition to the urgent HBP values, we stated the abnormal laboratory values in the letter, if present.

In the urgent notification letter, suspected disease name(s) and brief interpretation regarding the abnormal data were described. Concurrently, recommendation as to which type of medical care would be adequate for the participant was given. Representative letters of urgent notification for HBP are shown in Figure 4. These letters were delivered through the Japanese postal service, which took 1 to 2 days to arrive. As shown in Figure 2, the participants may receive the urgent notification letters within 1 week after return of the sphygmomanometer device to the community support centers ^(16), (17), (18), (19)^.

### Urgent notification cases for HBP

**Figure 5.**
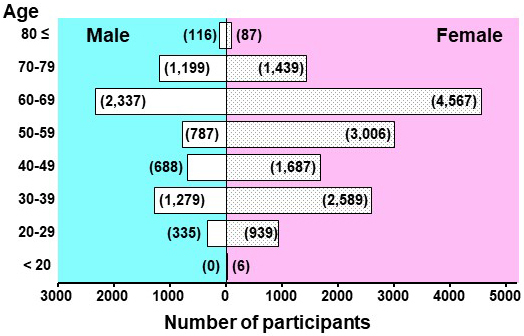
Numbers of participants and their gender distributions. As for the numbers of participants, two age peaks, 30’s and 60’s years old, were observed both in men and women. The numbers of the female participants were approximately twice more than that of the male participants. Approximately 40% of the participants were aged >60 years.

We instructed the 21,061 participants (14,320 women and 6,741 men) to measure their HBP (Figure 1 and 5). Because of the design of our cohort study, the numbers of male and female participants exhibited two peaks, one in the 30s and the other in the 60s. Approximately 40% of the participants were aged >60 years; thus, it is likely that some of the participants had high blood pressure. The frequency of HBP recording varied among the participants; some measured it regularly throughout the day and weeks, whereas others performed the measurements less frequently.

The criteria for the decision to whether send the urgent notification letter or not were established. The urgent notification letters were sent to the participants who had average HBP values of at least three measurements that met the criteria for hypertension. If the average was based on two or fewer measurements, even if it met the criteria for hypertensive emergency, we did not send urgent notification letters.

As the participants took the HBP monitoring device home and measured their blood pressure for 14 days, the blood pressure measurements were analyzed almost 3 weeks after the blood test at the community support centers (Figure 2). The blood test reports were already completed and stored in the ToMMo database. Therefore, laboratory test data with abnormal but not urgent results were included in the urgent notification letters for high blood pressure as needed (Figure 4).

#### i) Urgent notification for hypertensive emergency

The HBP measurement was started at the community support centers in October 2013. At the beginning (from October 2013 to May 2014), we returned urgent notification letters when the mean HBP value were met the criteria of Grade II hypertension equivalent or higher (blood pressure ≥ 160/100 mmHg), based on the 2009 Japanese Society of Hypertension (JSH2009) Guidelines ^(15)^. During this period, urgent notification letters were sent to 210 participants who had high blood pressure. Of these participants, 196 had Grade II hypertension (blood pressure >160/100 mmHg but <180/110 mmHg) and 14 had Grade III hypertension (blood pressure > 180/110 mmHg) according to the JSH2009 Guidelines.

After our urgent notification operation for approximately 9 months, we realized that notification of the regular health assessment report within 3 months is sufficient for notifying the risk of hypertension of Grade II participants. Therefore, in July 2014, we revised our criteria for the urgent notification of HBP values and decided to send urgent notification letters only to participants with Grade III hypertension. Consequently, we sent urgent notification letters to 46 participants afterward. In the end, a total of 256 participants out of 299 received the urgent notification letters.

#### ii) Associating findings with hypertension

In this study, we sent urgent notification letters to 256 participants, of whom 60 and 196 had Grades III and II hypertension, respectively. However, two participants withdrew from the study, leaving us with 254 participants, of whom 198 (78%) had additional abnormal findings in their laboratory test data (Table 1 and 2). The most frequently observed abnormality was related to glucose metabolism (n = 118). Renal dysfunction (n = 101) and abnormal serum lipid levels (n = 90) were also found. Many participants had multiple types of abnormal findings. Notably, the chi-squared or Fisher’s exact test for 2 × 2 contingency tables revealed a significant association between abnormal blood sugar level and abnormal lipid level (Table 2), which are suggestive of metabolic syndrome. As this is the first cross-sectional cohort survey we conducted, it is difficult to draw conclusions regarding the association between elevated blood pressure levels and abnormal laboratory test data.

#### iii) Effect of urgent notification on the participants’ behavior

As part of the ToMMo cohort program, we conducted annual questionnaire surveys on individuals who participated in the initial cross-sectional cohort survey ^(3), (6), (7), (8)^. We could analyze annual questionnaires related to the status of hypertension treatment from the 254 participants who received urgent notification letters (Table 3). We compared the questionnaires at the pre-survey with those after the urgent notification. We obtained responses to the questionnaires from 228 and 163 participants before and after sending the urgent notification letters, respectively. The pre-survey questionnaires indicated that 95 participants were already “under regular treatment” in the medical clinic before receiving the urgent notification letters. Notably, this number increased to 116 out of the 163 participants who responded to the questionnaires after the urgent notification letters (Table 3). Contrarily, the number of participants who reported “discontinuation of follow-up,” “doctor’s follow-up without medication,” or “never being pointed out” in response to inquiries about their hypertension status significantly decreased. Thus, our urgent notification letter appeared to have motivated the participants who were previously unconcerned about their blood pressure to seek medical attention. However, because of the inherent nature of the cohort studies, the number of participants who did not provide any response or feedback increased from 26 to 91.

**Table 3.** Effect of Urgent Notification on the Participants’ Behavior.

	Response to the questionnaire of all the participants^1^					No answer nor response	Total
	Under regular treatment	Discontinuation of follow-up	Lifestyle precaution	Doctor’s follow-up w/o medication	Never being pointed out		
Presurvey (n = 228)	95 (41.7%)^2^	15 (6.6%)	39 (17.1%)	24 (10.5%)	55 (24.1%)	26	254
Posturgent notification (n = 163)	116 (71.2%)	3 (1.8%)	21 (12.9%)	8 (4.9%)	15 (9.2%)	91
	Participants who responded to both questionnaires^3^						
Presurvey (n = 151)	68 (45.0%)^4^	7 (4.6%)	25 (16.6%)	14 (9.3%)	37 (24.5%)	NA^5^	151
Posturgent notification (n = 151)	107 (70.9%)	3 (2.0%)	20 (13.2%)	8 (5.3%)	13 (8.6%)

Answer to questionnaires regarding the treatment statues of hypertension from participants (n = 254) who received urgent notification letters of high systolic and/or diastolic blood pressure, >180 (some were >160; *see text*) and/or >95, respectively. The ^1^numbers of participants who responded to the ^1^questionnaires of the pre-survey and of the post-emergent notification are shown in the top two rows. The percentages of participants who responded (n = 228 and n = 163 for pre-survey and post-urgent notification, respectively) are presented in the parentheses. ^3^Number of participants who responded to both the pre-survey and the post-urgent notification are subgrouped, and their answers in the pre-survey and post-urgent notification are shown in the bottom two rows. The ^4^percentages of participants in the subgroup are presented in the parentheses. ^5^Not applicable.

Among the 254 participants who received urgent notification letters, 151 were divided into subgroups, namely, those who responded to the pre-survey questionnaire, those who responded to the post-urgent notification questionnaire, and those who responded to both questionnaires, and their behaviors toward hypertension were examined (Table 3). The number of participants who were “under regular treatment” increased from 68 to 107, whereas that of participants who reported “never being pointed out” significantly decreased from 37 to 13. There is an increasing trend for the regular treatment after the notification. However, the increase in the number of participants who were “under regular treatment” after the urgent notification did not exhibit statistical significance compared with total of other four behaviors analyzed using McNemar’s chi-squared test (*P* = 0.08).

## Discussion

Elucidating how the GEJE has affected the residents’ long-term health is a challenging issue, and it is particularly intriguing how the participants think about their health management after the earthquake. In the TMM project, we conducted health checkups at baseline and during the follow-up surveys and regularly returned the results to the participants. During the health checkups, we occasionally encountered extremely abnormal values, which prompted us to develop an urgent notification system. In this study, we described how we designed and operated the system, with special reference to the type of serious health issues that emerged and how participants responded to the urgent notification.

During the health checkups, we occasionally encountered highly abnormal HBP levels. In the developed urgent notification system, if high HBP levels are observed, our UNO staff verify the data and promptly issue urgent notification letters, advising the participants to seek immediate treatment. Simultaneously, we thoroughly investigated the impact of our urgent notification system on the promotion of the participants’ health consciousness. Intervention, such as the urgent notification in this study, into prospective cohort studies may not be a common standpoint of a standard regimen. However, we considered it ethically crucial to inform participants about their urgent health status, which may indicate serious illnesses, especially in population cohorts established after heavy disaster. We analyzed the efficacy of urgent notifications through questionnaires and follow-up surveys and found that most of the participants heed our recommendations and visited hospitals or clinics.

Because our participants are distributed relatively wide-range across middle to elderly individuals, which may introduce potential biases, we observed the predominance of metabolic syndrome-related signs in the blood tests. For instance, we frequently encountered abnormal blood sugar levels, which exhibited a significant correlation with hypertension (Table 2). This may be pertinent with the increase in metabolic issues in Miyagi prefecture, particularly after the GEJE ^(20)^. To ensure widespread awareness, we shared these findings with the residents and participants through various channels. We remained vigilant about assessing the potential long-term effects of our interventions.

Urgent notification of hypertension through HBP monitoring is the most frequently used intervention in our study. It is well known that large-scale disasters, such as the GEJE, can cause severe stress among residents and cause withdrawal from hypertension treatment ^(21)^, particularly during the acute phase, thus increasing the cases of hypertension ^(22), (23), (24), (25), (26)^. Insufficient medical care and inappropriate diet during the evacuation period may exacerbate blood pressure issues. In fact, examinations of disaster victims from various events, such as the Hanshin Awaji earthquake in 1995 ^(27), (28), (29)^, Central Italy Earthquake in 1998 ^(30)^, September 11 attacks in 2001 ^(31), (32)^, Mid-Niigata Earthquake in 2004 ^(33)^, Wenchuan Earthquake in 2009 ^(34)^, and L’Aquila Earthquake in 2009 ^(35)^, revealed that acute-phase disasters are often associated with increased incidence of hypertension and cardiovascular diseases.

Likewise, our cohort study was conducted in areas severely affected by the GEJE and tsunami. One of the aims of this study was to contribute to the maintenance of the residents’ health status. By comparing the results of this study with those of cohort studies conducted in other areas in Japan or worldwide, it may be possible to identify factor(s) that can mitigate health damage during disasters.

In the HBP measurement performed for 14 days, we instructed the participants to measure their HBP at least once in the morning and once in the evening. One measurement in the morning and one in the evening for 3 days are generally sufficient to estimate the reliable risk for hypertension and cardiovascular diseases ^(16), (17), (18), (19)^, indicating that at least 6 measurements are required. Moreover, most of the HBP studies and trials conducted so far evaluated the mean of all HBP measurements for 7 days ^(36)^. Our cohort generally used more than 10 times of measurement within the 14 days, thus providing the reliable risk estimate.

The ToMMo cohort studies aimed to contribute to the health maintenance of residents after disasters. Therefore, the cohort studies were conducted from the subacute to the chronic phase, as long-term or intense stress may cause transient hypertension to progress to an established condition. The urgent notification system proved beneficial as the participants became aware of their high blood pressure and subsequently underwent medical checkups. Of the participants who received the urgent notification letter, 41.7% were already on regular medical care, and this percentage increased to 71.2% after the urgent notification (Table 3), indicating that this system played an important role in motivating residents to seek medical care. For participants under hypertension treatment, urgent notification may serve as a useful message for their doctors to optimize the treatment approach.

In conclusion, our challenge into the urgent notifications revealed that cases of severe hypertension with metabolic syndrome are most predominant among recipients of the urgent notifications. These individuals will be in a serious condition without appropriate cares ^(37)^. However, more than half of those who received the urgent notification letters started visiting medical institutions. This notification intervention is ongoing in our cohort studies to assess its effect on health outcomes. The urgent notification system has proven effective in encouraging participants to take care of themselves under the guidance of medical professionals, contributing to the maintenance of good health in the community.

## Article Information

### Conflicts of Interest

None

### Sources of Funding

This work was supported, in part, by grants from the Reconstruction Agency; Ministry of Education, Culture, Sports, Science and Technology (MEXT); and Japan Agency for Medical Research and Development (AMED) under grant numbers JP 17 km0105001 and JP 21tm0124005.

### Acknowledgement

The authors greatly thank Mami Funata for collecting data and providing us the descriptive data with assistance and Drs. Osamu Tanabe, Yasuyuki Taki, Soichiro Toda, Takako Takai, Hiroaki Tomita, Akito Tsuboi, and Hiroshi Kawame for providing great support for the establishment of urgent notification system. We appreciate Professor Shizuo Fujiwara, Chuo University, the chairman of an Ethical, Legal, and Social Issues (ELSI) Committee for the Tohoku Medical Megabank Project, which consist of only external experts and aims to oversee the Steering Committee and review the various ethical, legal, and social issues. We thank the hospitals and/or clinics for providing medical care to the referred participants and local medical associations for medical cooperation. We thank the members of the Tohoku Medical Megabank Organization, including the Genome Medical Research Coordinators, and the office and administrative personnel for their assistance. We are grateful to everyone who participated in or worked for the cohort to make the studies possible. The complete list of members is available at https://www.megabank.tohoku.ac.jp/english/a220901/.

### Author Contributions

E.N. Kodama, M. Taira, H. Kiyomoto, S. Kuriyama, A. Hozawa, J. Sugawara, F. Nagami, J. Nakaya, H. Metoki, M. Sakaida, M. Kikuya, Y. Suzuki, K. Ito, K. Suzuki, S. Kure, N. Yaegashi, N. Fuse, R. Shimizu, and M. Yamamoto designed the study.

E.N. Kodama, M. Taira, H. Kiyomoto, T. Nakamura, S. Kuriyama, A. Hozawa, J. Sugawara, F. Nagami, A. Uruno, J. Nakaya, H. Metoki, M. Sakaida, M. Kikuya, Y. Suzuki, Y. Hamanaka, K. Suzuki, S. Kure, N. Fuse, and R. Shimizu performed data acquisition.

E.N. Kodama, M. Taira, H. Kiyomoto, T. Nakamura, S. Nagaie, A. Hozawa, F. Nagami, R. Shimizu, and M. Yamamoto analyzed and interpreted data.

E.N. Kodama, M. Taira, T. Nakamura, S. Nagaie, A. Hozawa, F. Nagami, N. Fuse, R. Shimizu, and M. Yamamoto drafted the manuscript or revised it critically.

E.N. Kodama and M. Taira contributed equally to this work.

### Approval by Institutional Review Board (IRB)

This study was approved by the Institutional Review Board of the Tohoku Medical Megabank Organization (approval number: 2022-4-047; approval date: 30 June 2022).
